# miR-27b-3p Exacerbates VCD-Induced KGN Cell Injury by Targeting PAPPA to Suppress IGF-1 Release and Inhibit the PI3K/AKT Pathway

**DOI:** 10.3390/genes17080966

**Published:** 2026-08-18

**Authors:** Manyu Zhang, Xiangyu Meng, Mengdi Shi, Pengling Ge

**Affiliations:** 1 Department of Pharmacology, School of Basic Medical Sciences, Heilongjiang University of Chinese Medicine, Harbin 150040, China; zmy057@hotmail.com; 2 Department of Medical History and Literature of TCM, School of Basic Medical Sciences, Heilongjiang University of Chinese Medicine, Harbin 150040, China; mengxilun@163.com; 3 Department of Medical Affairs, The Second Affiliated Hospital of Heilongjiang University of Chinese Medicine, Harbin 150040, China; s8130239@163.com

**Keywords:** miR-27b-3p, PAPPA, IGF-1/PI3K/AKT pathway, ovarian granulosa cells, premature ovarian insufficiency (POI), VCD-induced injury model

## Abstract

Background/Objectives: While currently termed premature ovarian insufficiency (POI), premature ovarian failure (POF) remains a prominent driver of female infertility with a molecular pathogenesis that is still lacking comprehensive clarification. In in vitro studies, the pathology of POI is commonly simulated through a well-characterized model involving VCD (4-vinylcyclohexene diepoxide)-mediated cytotoxicity within KGN-derived human granulosa cells. However, the key regulatory molecular networks involved in this process are still poorly characterized. Although microRNAs (miRNAs) have emerged as critical regulators in ovarian function decline, the specific role and underlying mechanism of miR-27b-3p in POI remain elusive. Methods: A VCD-induced KGN cell injury model was established by treating cells with 1.0 mM VCD for 24 h. Cell viability, apoptosis rate, and miR-27b-3p expression were assessed by CCK-8 assay, flow cytometry, and RT-qPCR, respectively. Overexpression and targeted suppression of miR-27b-3p were achieved by introducing its specific mimics and inhibitors, respectively. Target identification was conducted via bioinformatic prediction, EdU incorporation, Western blot, and dual-luciferase reporter assays. Functional rescue experiments were carried out by co-transfection with a PAPPA-overexpressing plasmid (oe-PAPPA). IGF-1 secretion was quantified by ELISA, and phosphorylation of IGF1R and AKT was analyzed by Western blot to determine whether miR-27b-3p modulates cellular phenotypes via the PAPPA–IGF-1–PI3K/AKT axis. Exogenous IGF-1 supplementation was further applied to confirm pathway dependence. Results: VCD treatment dose-dependently restrained cellular growth and stimulated apoptotic pathways in KGN cells; paralleling these phenotypic changes, miR-27b-3p abundance was remarkably increased. Ectopic expression of miR-27b-3p exacerbated VCD-induced growth inhibition and apoptosis, whereas its inhibition conferred cytoprotective effects. Through the integration of computational predictions and dual-luciferase reporter systems, PAPPA was definitively established as a direct downstream target of miR-27b-3p. miR-27b-3p negatively regulated both PAPPA mRNA and protein levels, thereby impairing PAPPA-mediated cleavage of IGF-binding proteins (e.g., IGFBP4) and subsequent release of free IGF-1. This led to reduced IGF-1 secretion and significantly diminished phosphorylation of IGF1R and AKT. Remarkably, PAPPA overexpression effectively reversed the detrimental effects of miR-27b-3p, and exogenous IGF-1 supplementation similarly attenuated miR-27b-3p–mediated proliferation arrest and pro-apoptotic phenotypes. Conclusions: This study uncovers a novel pathogenic mechanism whereby miR-27b-3p exacerbates VCD-induced granulosa cell injury by directly targeting PAPPA, suppressing IGF-1 release, and consequently inhibiting the PI3K/AKT pro-survival signaling pathway. A novel perspective on the fundamental basis of POI is established by this research, which further posits therapeutic manipulation of the miR-27b-3p/PAPPA/IGF-1 module as a prospective treatment for disrupted ovarian function.

## 1. Introduction

Diagnosed primarily by the premature cessation of gonadal function before the fourth decade of life, premature ovarian insufficiency (POI, previously known as POF) is typified by absent menses, inadequate estrogen levels, and sterility. Consequently, this endocrine disorder severely disrupts the reproductive trajectories and long-term health landscape of women in their childbearing years [1,2,3]. Although genetic, autoimmune, and iatrogenic etiologies have been extensively documented [4,5], the underlying molecular mechanisms remain incompletely elucidated, particularly with respect to the systemic regulatory networks governing granulosa cell (GC) dysfunction—a central pathological feature of POI.

4-Vinylcyclohexene diepoxide (VCD), an environmental chemical toxin, selectively targets granulosa cells in primordial and primary follicles, making it a robust, reproducible in vitro model for recapitulating POI-associated follicular atresia [6,7]. Accumulating evidence characterizes non-coding transcripts, notably microRNAs (miRNAs), as critical orchestrators of follicular development, steroid hormone synthesis, and granulosa cell survival. Consequently, their exact involvement in the molecular etiology of POI has become a focal point of contemporary investigations. [8]. For instance, miR-132-3p suppresses GC apoptosis by targeting FOXO1 [9]; miR-383-5p promotes GC apoptosis via CIRP repression and subsequent PI3K/AKT pathway dysregulation [10] miR-6881-3p modulates GC apoptosis through SMAD4 targeting, contributing to diminished ovarian reserve [11]; and miR-361-5p preserves mitochondrial function by regulating SLC25A24, thereby alleviating GC dysfunction under conditions of reduced ovarian reserve [12]. Notably, the role of miR-27b-3p in ovarian physiology remains controversial: one study reported its upregulation in polycystic ovary syndrome (PCOS), where it inhibits GC proliferation and promotes apoptosis, exerting pro-injury effects [13]. In contrast, a distinct study revealed that miR-27b-3p is exceedingly abundant within exosomes secreted by human umbilical cord mesenchymal stem cells (hUSC-Exo). Through this delivery route, the microRNA effectively curtails GC apoptosis and exerts a protective effect against POI [14]. However, its direct target(s) and downstream molecular mechanisms remain undefined.

Insulin-like growth factor-1 (IGF-1), a critical local paracrine/autocrine factor in the ovary, binds to IGF1R to activate the PI3K/AKT signaling cascade, thereby sustaining GC survival, proliferation, and steroidogenesis [15,16]. Pregnancy-associated plasma protein A (PAPPA), a metalloproteinase, serves as a master regulator of IGF-1 bioavailability by specifically cleaving IGF-binding proteins (e.g., IGFBP4), thereby releasing free IGF-1 and amplifying its signaling potency [17]. Importantly, PAPPA expression is markedly upregulated during late follicular development; Pappa knockout mice exhibit follicular arrest and anovulation [18], while human studies consistently report significantly reduced PAPPA levels in follicular fluid from POI patients [19]. Clinical analyses further reveal that the dynamic decline in PAPPA activity at ovulation, coupled with consequent IGF-1 signaling suppression, acts as a key switch triggering GC proliferation arrest and luteinization [17]. Moreover, in Controlled ovarian stimulation protocols where follicular development is fully suppressed (programmed cycles, PC), significantly lower early-pregnancy maternal PAPPA and IGF-1 concentrations correlate with increased risk of placental insufficiency [20]. Collectively, these findings firmly establish the PAPPA–IGF-1 axis as a central regulator of human follicular function and integrity.

Given this context, we hypothesized that miR-27b-3p may contribute to POI pathogenesis by targeting PAPPA and disrupting IGF-1-mediated survival signaling in granulosa cells. To test this hypothesis, we used VCD-treated KGN cells, a well-established human ovarian granulosa-like cell model, to investigate the expression and function of miR-27b-3p and its potential regulatory relationship with PAPPA. We further examined whether the miR-27b-3p/PAPPA axis affects IGF-1 release and downstream IGF1R/AKT signaling, as well as granulosa cell proliferation and apoptosis. These analyses were designed to clarify the potential role and underlying molecular mechanism of miR-27b-3p in granulosa cell dysfunction associated with POI.

## 2. Materials and Methods

### 2.1. Cell Culture and Treatment

We acquired the KGN cells—an immortalized human ovarian granulosa-like tumor line—directly from Shanghai SaiBaikang Biotechnology Co., Ltd., (Shanghai, China). The basal culture environment utilized DMEM/F-12 (Genom Biotech, Hangzhou, China) enriched with 10% fetal bovine serum (Tianhang Biotechnology, Hangzhou, China). Cell propagation was carried out under stable conditions within a humidified 5% CO_2_ atmosphere at 37 °C. When the cells reached approximately 80% confluence, they were routinely digested and passaged or plated using 0.25% trypsin-EDTA. To determine the optimal VCD concentration for inducing granulosa cell injury, KGN cells were treated with 0, 30, 60, 90 μM, or 0.5, 1.0, 1.5, 3.0 mM 4-vinylcyclohexene diepoxide (VCD; Sigma-Aldrich, St. Louis, MO, USA) for 24 h. Based on CCK-8 and flow cytometry apoptosis assays, 1.0 mM VCD for 24 h induced significant proliferation inhibition and apoptosis elevation while retaining sufficient viability for subsequent transfection. Therefore, this condition was adopted to establish the VCD-induced injury model in all subsequent experiments. KGN cells were divided into six groups: the Control group, the VCD group, the VCD + NC mimic group, the VCD + miR-27b-3p mimic group, the VCD + NC inhibitor group, and the VCD + miR-27b-3p inhibitor group. The Control group consisted of untransfected KGN cells without VCD exposure. The VCD group consisted of untransfected KGN cells treated with 1.0 mM VCD for 24 h. For the transfection groups, KGN cells were transfected with miR-27b-3p mimics, miR-27b-3p inhibitors, or the corresponding negative controls at a final concentration of 50 nM using Lipofectamine™ 3000. The transfection mixture was retained, and the cells were cultured for 24 h after transfection. Subsequently, 1.0 mM VCD was added directly to the culture medium, and the cells were incubated for an additional 24 h. Thus, VCD treatment was initiated 24 h after transfection. Cells were harvested after VCD exposure for subsequent functional and molecular analyses.

### 2.2. Plasmids and Transfection

RiboBio (Guangzhou, China) custom-synthesized the miR-27b-3p mimics, inhibitors, alongside their parallel negative controls. Similarly, the PAPPA-upregulating plasmid (oe-PAPPA) and its corresponding empty backbone (Vector) were engineered by ELK Biotechnology (Shanghai, China). For cellular delivery, we employed Lipofectamine™ 3000 (Invitrogen, Carlsbad, CA, USA), strictly following the supplier’s guidelines. Working doses were uniform: 50 nM for all RNA oligonucleotides and 0.2 μg per well for plasmid DNA. Following a 6 h initial exposure, the lipid-reagent mixture was discarded and replenished with fresh medium, permitting the cultures to stabilize for another 24 h.

### 2.3. Real-Time Quantitative Reverse Transcription PCR (RT-qPCR)

Total RNA was extracted from KGN cells using 1 mL of TRIpure™ Reagent (ELK Biotechnology, Shanghai, China). RNA integrity and purity were assessed using an ND-100 micro-spectrophotometer (Miulab, Hangzhou, China). First-strand cDNA was generated leveraging the EntiLink™ Synthesis Kit (ELK Biotechnology, Shanghai, China). Specifically, stem-loop oligonucleotides were applied for miR-27b-3p reverse transcription, while Random Primers N6 were deployed for PAPPA mRNA. Subsequent quantitative PCR assessments took place on a QuantStudio™ 6 Flex platform (Life Technologies, Carlsbad, CA, USA) powered by the EnTurbo™ SYBR Green SuperMix (ELK Biotechnology, Shanghai, China). For data quantification, the $2^{-\Delta \Delta Ct}\;$ algorithm was applied. GAPDH and U6 were adopted as internal reference baselines to normalize PAPPA and miR-27b-3p abundances, respectively. All sequence details for the primers are inventoried in Table 1.

### 2.4. Western Blotting

Whole-cell lysates from KGN cultures were harvested using RIPA lysis buffer (Beyotime, Shanghai, China). Protein yields were subsequently quantified utilizing a BCA Protein Assay Kit (Pierce, Thermo Fisher Scientific, Appleton, WI, USA). Following standardization, 40 μg aliquots of protein were resolved via 10–12% SDS-PAGE and electroblotted onto PVDF membranes (Millipore, Billerica, MA, USA). To saturate non-specific binding sites, blots were immersed in 5% skim milk—or 5% BSA when evaluating phosphorylated targets—for 1 h at ambient temperature. Finally, the membranes underwent an overnight antibody probing phase at 4 °C utilizing the following primary antibodies:cleaved caspase-3 (1:500, AF7022, 17 kDa), PAPPA (1:500, 18095-1-AP, 180 kDa), p-IGF1R (1:500, AP0367, 95 kDa), IGF1R (1:1000, #3027, 95 kDa), p-AKT (1:1000, #4060, 60 kDa), AKT (1:3000, #4691, 60 kDa), and GAPDH (1:10,000, ab181602, 37 kDa). After three 5 min washes with TBST, membranes were incubated with HRP-conjugated goat anti-rabbit secondary antibody (1:10,000, AS1107, ASPEN, Shanghai, China) at room temperature for 30 min. Following four TBST washes, signals were visualized using an ECL chemiluminescence kit (AS1059, ASPEN, Shanghai, China) and quantified by ImageJ (version 1.53k, National Institutes of Health, Bethesda, MD, USA). The relative abundance of target proteins was standardized against GAPDH as an internal control, followed by the determination of the phosphorylated-to-total protein ratios for both IGF1R and AKT. Each assay was carried out utilizing three technical replicates and validated across three independent biological trials.

### 2.5. Flow Cytometric Analysis of Apoptosis

Following a washing step with PBS, 2 × 10^5^ KGN cells were plated into each well of a 6-well culture dish. To evaluate apoptosis, the cells were labeled with the Annexin V-FITC/PI Apoptosis Detection Kit (BD Biosciences, San Jose, CA, USA) and subsequently maintained on ice. Flow cytometric acquisition was executed utilizing a CytoFLEX system (Beckman Coulter, Brea, CA, USA), and the resulting data were evaluated via FlowJo v7.6 software (Treestar, Ashland, OR, USA). The entire protocol was subjected to three independent biological replicates.

### 2.6. Cell Proliferation Assay (CCK-8)

KGN cells were plated into 96-well microplates and underwent transfection upon reaching a cell density of 40–60%. To evaluate cell viability at specified intervals (24, 48, and 72 h after transfection), each specific well received 10 μL of the CCK-8 reagent (Vazyme, Nanjing, China). Following an additional 2 h incubation period, the optical density (OD) at 450 nm was recorded utilizing a BioTek microplate reader (Winooski, VT, USA).

### 2.7. EdU Incorporation Assay

KGN cells were distributed into 96-well plates and underwent transfection upon achieving 40–60% confluency. To evaluate DNA synthesis 24 h post-transfection, the samples were labeled using the Cell-Light™ EdU Apollo567 In Vitro Kit (RiboBio, Guangzhou, China) in accordance with the manufacturer’s directions. Microscopic imaging was performed with a Leica DMi8 inverted fluorescence microscope (Leica Microsystems, Wetzlar, Germany) to acquire three randomly selected fields per well. The enumeration of specifically EdU-incorporated cells versus the total cell population was subsequently executed via ImageJ (version 1.53k, National Institutes of Health, Bethesda, MD, USA).

### 2.8. Dual-Luciferase Reporter Assay

To prepare the reporter constructs, the unaltered 3′UTR of PAPPA was inserted into a pGL6 backbone (ELK Biotechnology, Shanghai, China), yielding pGL6-PAPPA-3′UTR-WT. Concurrently, a mutated version (pGL6-PAPPA-3′UTR-Mut) was generated by specifically altering the theoretical miR-27b-3p recognition motif, while the unmodified pGL6 plasmid functioned as the negative control (NC). For transient expression, 293T cells cultured in 24-well dishes were allowed to reach 30–50% confluence. These cells were subsequently cotransfected utilizing Lipofectamine^®^ 2000 (Invitrogen, Carlsbad, CA, USA) with a defined mixture: 0.2 μg of the respective pGL6 construct, 0.02 μg of the pRL-TK internal control, and 50 nM miR-27b-3p mimics (or NC). Twenty-four hours post-treatment, luminescent signals were quantified via a Spark™ 10M Multimode Microplate Reader (Tecan, Männedorf, Switzerland) using the Dual-Luciferase Reporter Assay Kit (Beyotime, Shanghai, China). Final reporter activation was determined by normalizing the Firefly luminescence units to those of the Renilla internal reference. Triplicate wells were assigned for all conditions across three distinct biological replicates.

### 2.9. Statistical Analysis

Quantitative results are expressed as the mean ± standard deviation (SD). To evaluate differences between two distinct groups, the independent Student’s *t*-test was employed. When analyzing three or more cohorts, a one-way analysis of variance (ANOVA) followed sequentially by Tukey’s post hoc test was utilized. Statistical significance was established at a threshold of *p* < 0.05. The computational analyses were executed via IBM SPSS Statistics version 26.0 (Armonk, NY, USA), whereas data visualization and charting were accomplished using GraphPad Prism 10 (San Diego, CA, USA).

## 3. Results

### 3.1. Establishment of the VCD-Induced KGN Cell Injury Model and Upregulation of miR-27b-3p

**Figure 1:**
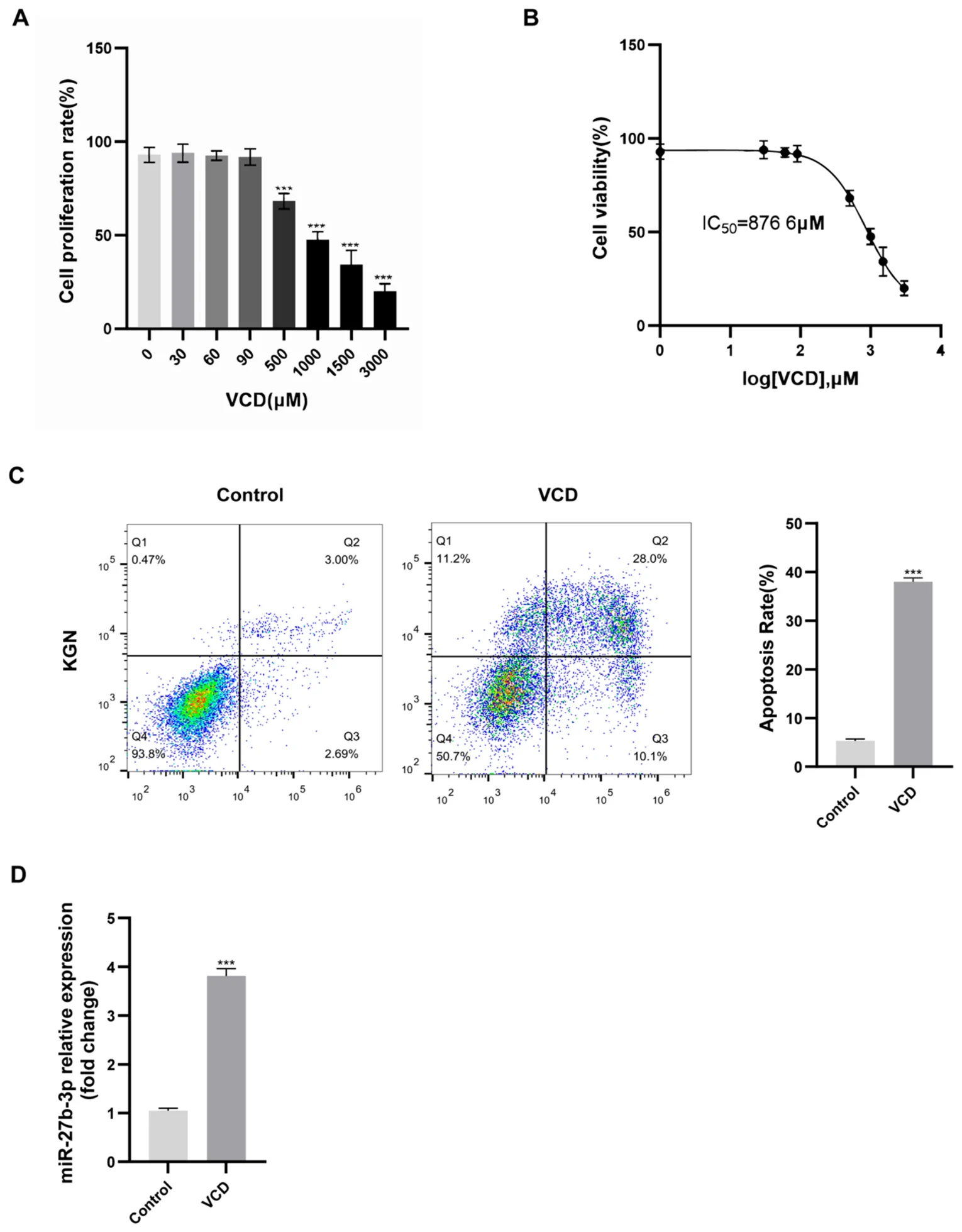
Establishment of the VCD-induced KGN cell injury model and upregulation of miR-27b-3p. (**A**) CCK-8 assay showing dose-dependent suppression of KGN cell proliferation after 24 h of VCD treatment (0–3000 μM). *** *p* < 0.001 vs. 0 μM group. (**B**) Nonlinear regression analysis yielding an IC_50_ of 876.6 μM for VCD. (**C**) Flow cytometric analysis of apoptosis in KGN cells treated with 1.0 mM VCD for 24 h. *** *p* < 0.001 vs. Control. In subfigure (**C**), the colors indicate event density rather than distinct cell populations or experimental groups, with warmer colors representing a higher cell density. (**D**) RT-qPCR analysis of miR-27b-3p expression in VCD-treated KGN cells. *** *p* < 0.001 vs. Control. The results are expressed as averages with their corresponding standard deviations (mean ± SD) based on biological triplicates.

A CCK-8 assay was employed to evaluate the survival rate of KGN cells, aiming to construct an in vitro model of VCD-induced damage and identify the most appropriate chemical dosage. Specifically, the cellular cultures were exposed to a concentration gradient of VCD (ranging from 0 to 3000 μM) for a duration of 24 h. As shown in Figure 1A, low-dose VCD (30–90 μM) did not significantly affect cell viability compared to the untreated Control. However, treatment with ≥0.5 mM VCD induced a significant, dose-dependent reduction in cell proliferation (*p* < 0.001). Nonlinear regression analysis revealed an IC_50_ of 876.6 μM for VCD (Figure 1B), indicating clear dose-dependent cytotoxicity at ≥0.5 mM. Based on these data, 1.0 mM VCD (≈IC_50_) was selected as the standard modeling condition. Following the verification of apoptosis-dependent cytotoxicity via Annexin V-FITC/PI flow cytometry, we observed notable changes. Notably, VCD administration (1.0 mM for 24 h) profoundly amplified the overall proportion of apoptotic cells compared directly against the mock-treated baseline (*p* < 0.001; Figure 1C). These results confirm that 1.0 mM VCD for 24 h establishes a robust, reproducible in vitro injury model in KGN cells—inducing pronounced yet sublethal cytotoxicity and apoptosis while preserving sufficient viable cells for downstream transfection experiments. Consequently, this condition was uniformly applied in all subsequent assays. Subsequently, quantitative real-time PCR (RT-qPCR) was employed to evaluate miR-27b-3p transcript levels across untreated and VCD-exposed (1.0 mM, 24 h) KGN cells. This step aimed to elucidate the possible contribution of this microRNA to the pathogenesis of POI. Indicating its likely participation in VCD-driven granulosa cell damage, miR-27b-3p exhibited a robust increase in its expression levels within the experimental group (*p* < 0.01; Figure 1D).

### 3.2. miR-27b-3p Exacerbates VCD-Induced Proliferation Inhibition and Apoptosis in KGN Cells

**Figure 2:**
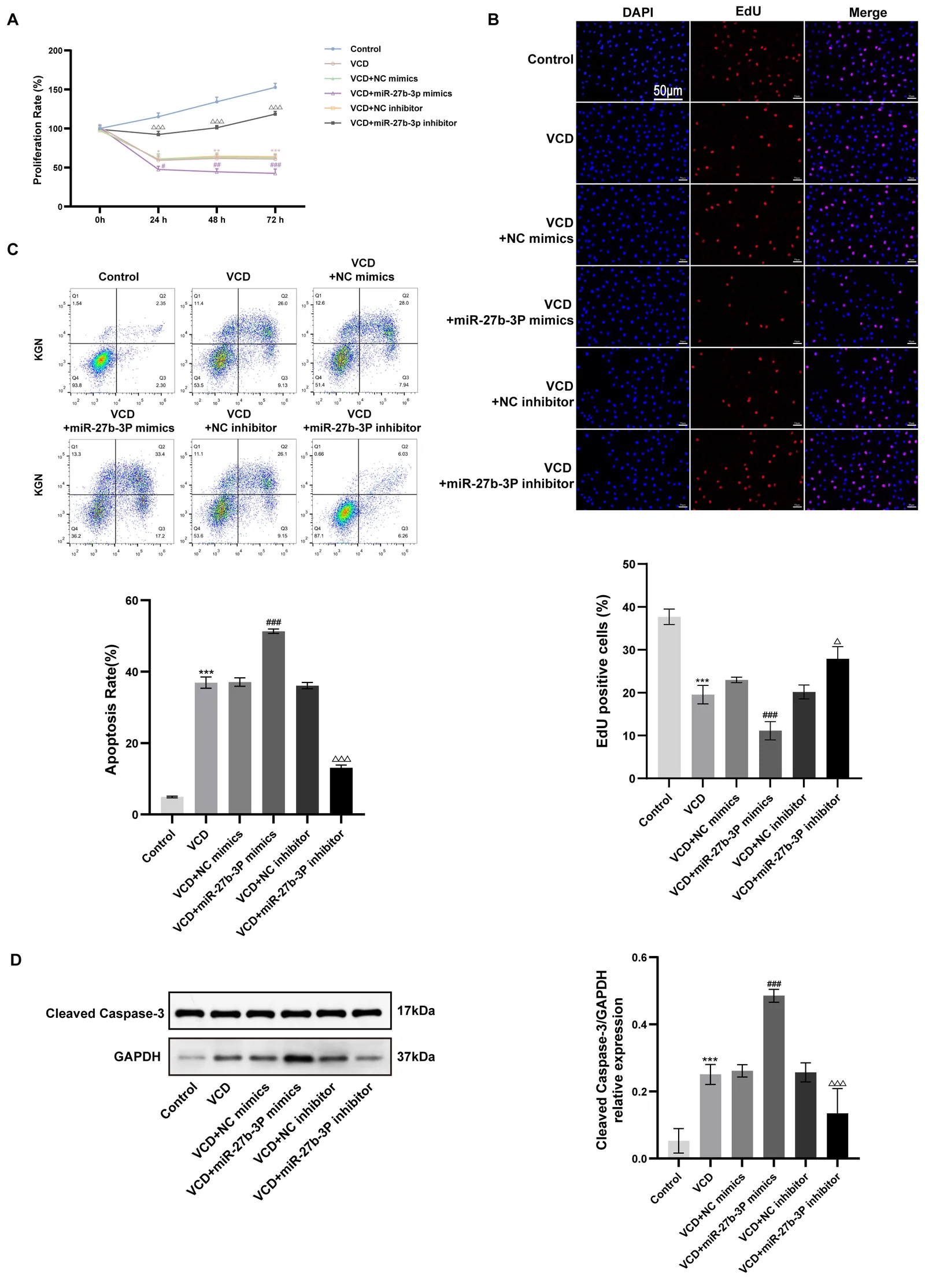
Aggravation of VCD-mediated anti-proliferative and pro-apoptotic effects by miR-27b-3p in KGN cells. The in vitro model was established by exposing KGN cells to 1.0 mM VCD concurrently with the introduction of miR-27b-3p mimics, inhibitors, or scrambled negative controls (NC). (**A**) CCK-8 assay showing cell proliferation at 0, 24, 48, and 72 h. (**B**) Representative EdU staining images (EdU^+^ cells: red; DAPI^+^ nuclei: blue). Scale bar = 50 μm (×200). (**C**) Flow cytometric apoptosis analysis and representative dot plots. In subfigure (**C**), the colors indicate event density rather than distinct cell populations or experimental groups, with warmer colors representing a higher cell density. (**D**) Western blot analysis of cleaved caspase-3 expression normalized to GAPDH. Data represent mean ± SD.* *p* < 0.05, ** *p* < 0.01, *** *p* < 0.001 vs. Control; ^#^ *p* < 0.05, ^##^ *p* < 0.01, ^###^ *p* < 0.001 vs. VCD + NC mimics; ^△^ *p* < 0.05, ^△△△^ *p* < 0.001 vs. VCD + NC inhibitor.

To elucidate the specific regulatory impact of miR-27b-3p during VCD-elicited damage, we subjected KGN cells to concurrent 1.0 mM VCD exposure and transfection with specific miR-27b-3p mimics, inhibitors, or corresponding scrambled controls (NC). CCK-8 and EdU assays collectively demonstrated that miR-27b-3p overexpression significantly aggravated VCD-induced proliferation suppression, whereas miR-27b-3p inhibition markedly attenuated it (*p* < 0.001; Figure 2A,B). Flow cytometry and Western blotting further confirmed that miR-27b-3p overexpression elevated apoptosis rate and upregulated cleaved caspase-3 expression, while its inhibition exerted protective effects (*p* < 0.001; Figure 2C,D). Collectively, miR-27b-3p promotes VCD-induced KGN cell injury by concurrently suppressing proliferation and enhancing apoptosis.

### 3.3. PAPPA Is a Direct Target of miR-27b-3p

**Figure 3:**
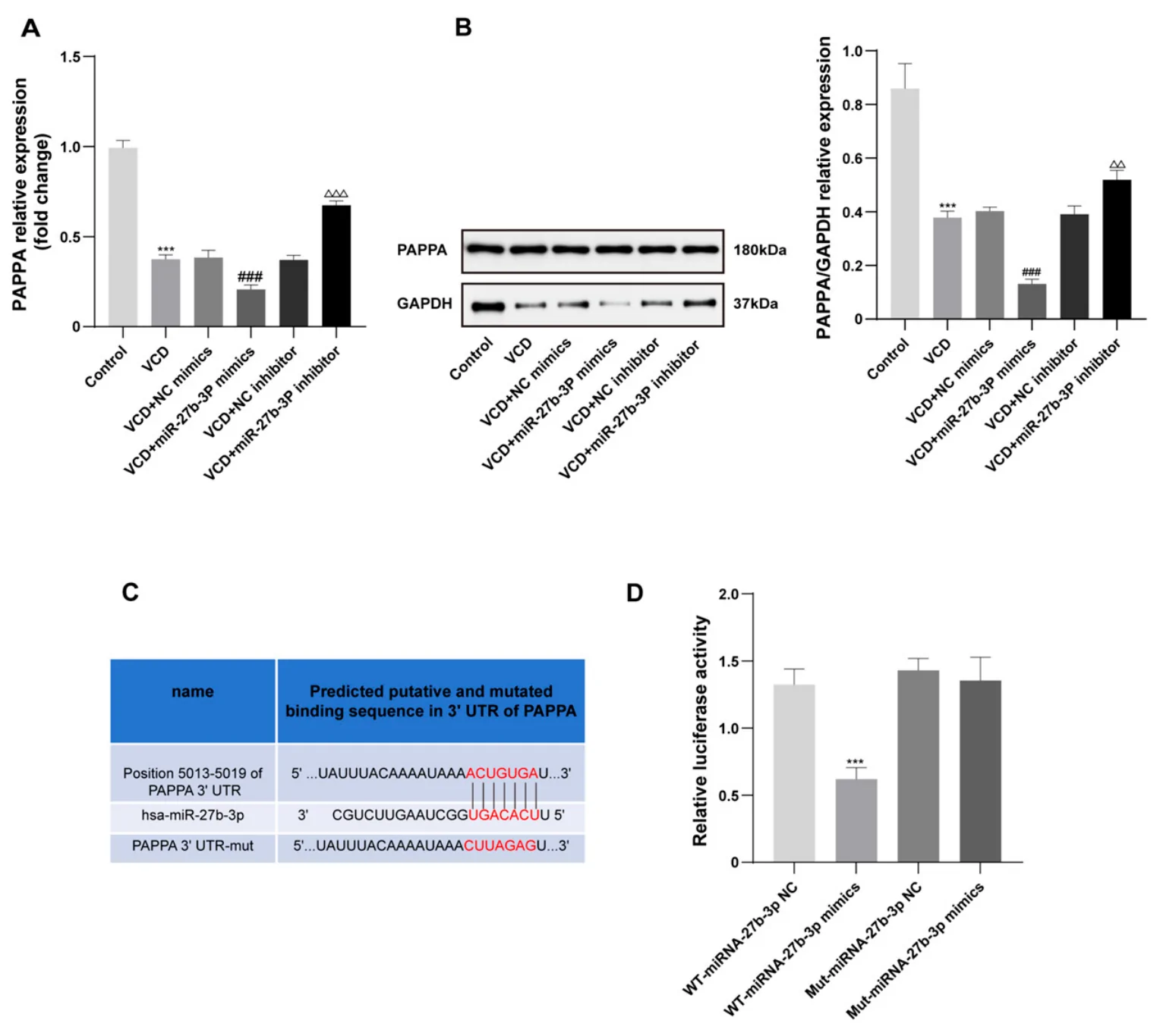
PAPPA is a direct target of miR-27b-3p in KGN cells. (**A**) RT-qPCR analysis of PAPPA mRNA levels after transfection. (**B**) Western blot analysis of PAPPA protein expression. (**C**) Predicted miR-27b-3p binding site in the PAPPA 3′UTR (WT) and its mutated sequence (Mut). (**D**) Dual-luciferase reporter assay in 293T cells. Data represent mean ± SD. *** *p* < 0.001 vs. Control; ^###^ *p* < 0.001 vs. VCD + NC mimics; ^△△^ *p* < 0.05, ^△△△^ *p* < 0.001 vs. VCD + NC inhibitor.

In silico screening utilizing the TargetScan algorithm revealed PAPPA as a potential downstream target of miR-27b-3p (Figure 3C). For empirical verification, KGN cells under VCD challenge were introduced to miR-27b-3p mimics or inhibitors. RT-qPCR revealed that miR-27b-3p overexpression significantly downregulated PAPPA mRNA, whereas its inhibition upregulated it (*** *p* < 0.001; Figure 3A). Western blotting further confirmed corresponding decreases and increases in PAPPA protein levels (*** *p* < 0.001; Figure 3B), indicating negative regulation of PAPPA by miR-27b-3p. To experimentally substantiate this specific binding, a dual-luciferase reporter system was utilized. Specifically, 293T cells were transiently co-transfected with pGL6 vectors harboring either the wild-type (WT) or mutant (Mut) 3′-UTR of PAPPA, alongside miR-27b-3p mimics or control oligos. The introduction of miR-27b-3p mimics markedly attenuated the luminescent signal from the wild-type construct (*** *p* < 0.001; Figure 3D). However, targeted mutagenesis of the putative recognition sequence completely reversed this inhibitory effect. Collectively, these findings support PAPPA as a direct post-transcriptional target of miR-27b-3p. The regulatory relationship was observed in VCD-treated KGN cells, while direct binding to the PAPPA 3′-UTR was validated in 293T cells.

### 3.4. miR-27b-3p Aggravates VCD-Induced KGN Cell Injury by Targeting PAPPA

**Figure 4:**
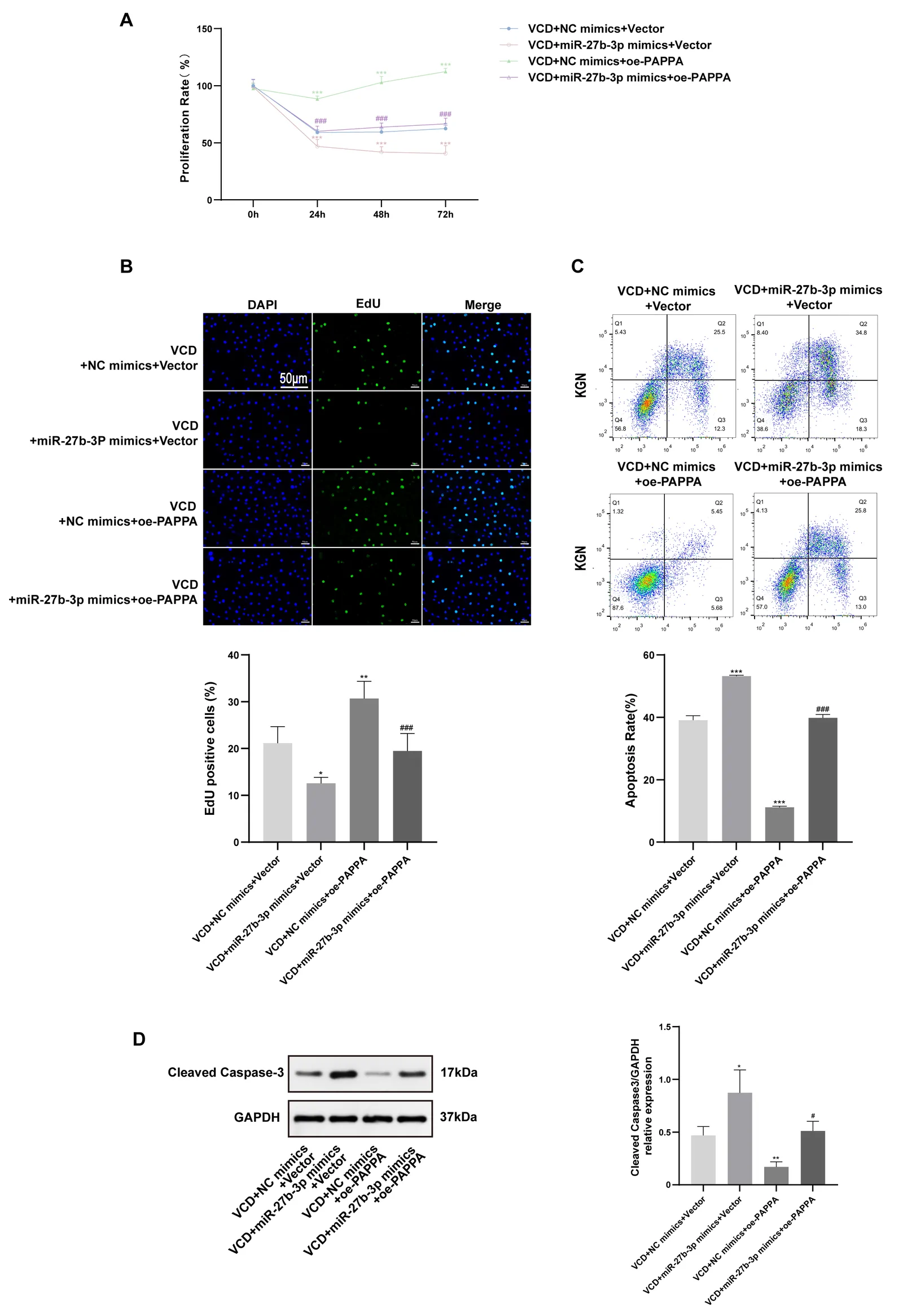
miR-27b-3p exacerbates VCD-induced KGN cell injury by targeting PAPPA. Phenotypic recovery evaluations in VCD-exposed KGN models following the concurrent delivery of miR-27b-3p mimics alongside PAPPA-expressing or control constructs. (**A**) CCK-8 proliferation curve over 72 h. (**B**) EdU staining and quantification. Scale bar = 50 μm (×200). (**C**) Flow cytometric apoptosis analysis. In subfigure (**C**), the colors indicate event density rather than distinct cell populations or experimental groups, with warmer colors representing a higher cell density. (**D**) Western blot of cleaved caspase-3 normalized to GAPDH. Data represent mean ± SD. * *p* < 0.05, ** *p* < 0.01, *** *p* < 0.001 vs. VCD + NC mimics + Vector; ^#^ *p* < 0.05, ^###^ *p* < 0.001 vs. VCD + miR-27b-3p mimics + Vector.

To dissect the functional interplay between miR-27b-3p and PAPPA, rescue experiments were conducted by co-transfecting miR-27b-3p mimics with oe-PAPPA or empty vector (Vector). Evaluations of cell growth via CCK-8 and EdU methodologies revealed that the singular introduction of miR-27b-3p mimics severely curtailed proliferative capacity (*p* < 0.001). Conversely, the independent overexpression of PAPPA exerted a dramatic stimulatory effect on cell expansion (*p* < 0.001; Figure 4A,B). Conversely, miR-27b-3p overexpression increased apoptosis and cleaved caspase-3 expression, while oe-PAPPA exerted potent anti-apoptotic effects (*p* < 0.001; Figure 4C,D). Critically, co-transfection of miR-27b-3p mimics with oe-PAPPA significantly reversed both the proliferation blockade and pro-apoptotic phenotype induced by miR-27b-3p (*p* < 0.001; Figure 4A–D). These findings establish PAPPA as a critical protective factor in the VCD-induced injury model and confirm that miR-27b-3p exacerbates granulosa cell damage primarily via direct suppression of PAPPA.

### 3.5. miR-27b-3p Suppresses IGF-1 Release and Inhibits PI3K/AKT Signaling via PAPPA Targeting

**Figure 5:**
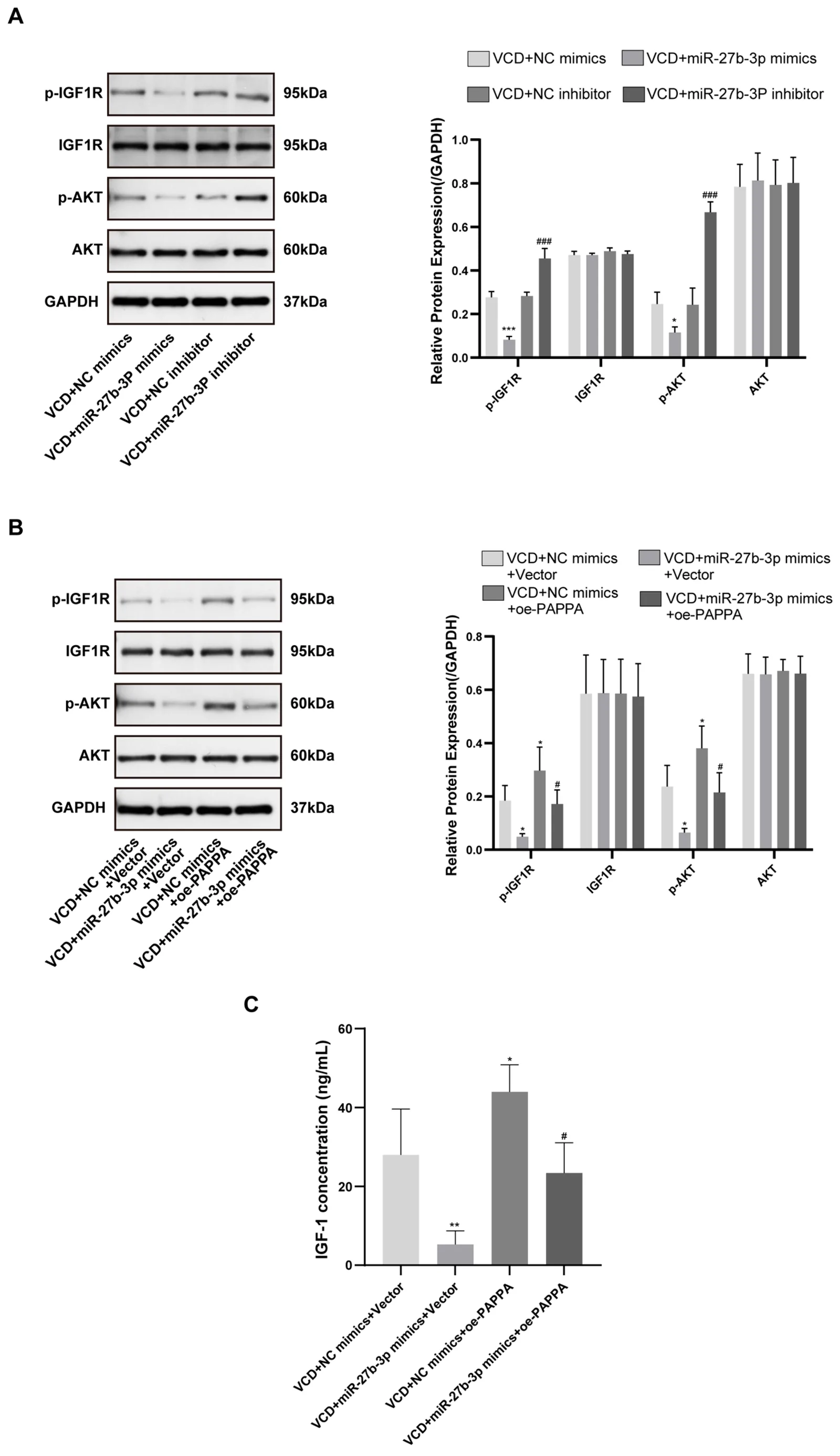
miR-27b-3p suppresses IGF-1 release and inhibits PI3K/AKT signaling via PAPPA targeting. (**A**) Western blot analysis of p-IGF1R, IGF1R, p-AKT, and AKT in VCD-injured KGN cells after miR-27b-3p modulation. * *p* < 0.05, *** *p* < 0.001 vs. VCD + NC mimics; ^###^ *p* < 0.001 vs. VCD + NC inhibitor. (**B**) Rescue of p-IGF1R/p-AKT by oe-PAPPA. * *p* < 0.05 vs. VCD + NC mimics + Vector; ^#^ *p* < 0.05 vs. VCD + miR-27b-3p mimics + Vector. (**C**) ELISA quantification of free IGF-1 in culture supernatants. * *p* < 0.05, ** *p* < 0.01 vs. VCD + NC mimics + Vector; ^#^ *p* < 0.05 vs. VCD + miR-27b-3p mimics + Vector. Data represent mean ± SD.

Aiming to elucidate the regulatory influence of miR-27b-3p upon the IGF-1/PI3K/AKT cascade, bidirectional modulation assays including both overexpression and silencing approaches were implemented. Protein-level validations demonstrated an inverse regulatory pattern: miR-27b-3p surplus profoundly impaired IGF1R and AKT activation (*p* < 0.001), whereas its depletion exerted a stimulatory effect on their phosphorylation, with total protein baselines remaining completely stable (Figure 5A). Given PAPPA’s role in liberating free IGF-1 via IGFBP cleavage, ELISA revealed that miR-27b-3p overexpression drastically reduced IGF-1 secretion in conditioned medium; this effect was fully rescued by oe-PAPPA co-transfection (*p* < 0.001; Figure 5C). Consistently, oe-PAPPA restored p-IGF1R and p-AKT levels suppressed by miR-27b-3p (*p* < 0.001; Figure 5B). Together, these data confirm that miR-27b-3p inhibits the IGF-1/PI3K/AKT survival pathway by directly targeting PAPPA, thereby limiting bioavailable IGF-1 in the granulosa cell microenvironment.

### 3.6. miR-27b-3p-Mediated Phenotypic Effects Are Dependent on IGF-1/PI3K/AKT Signaling

**Figure 6:**
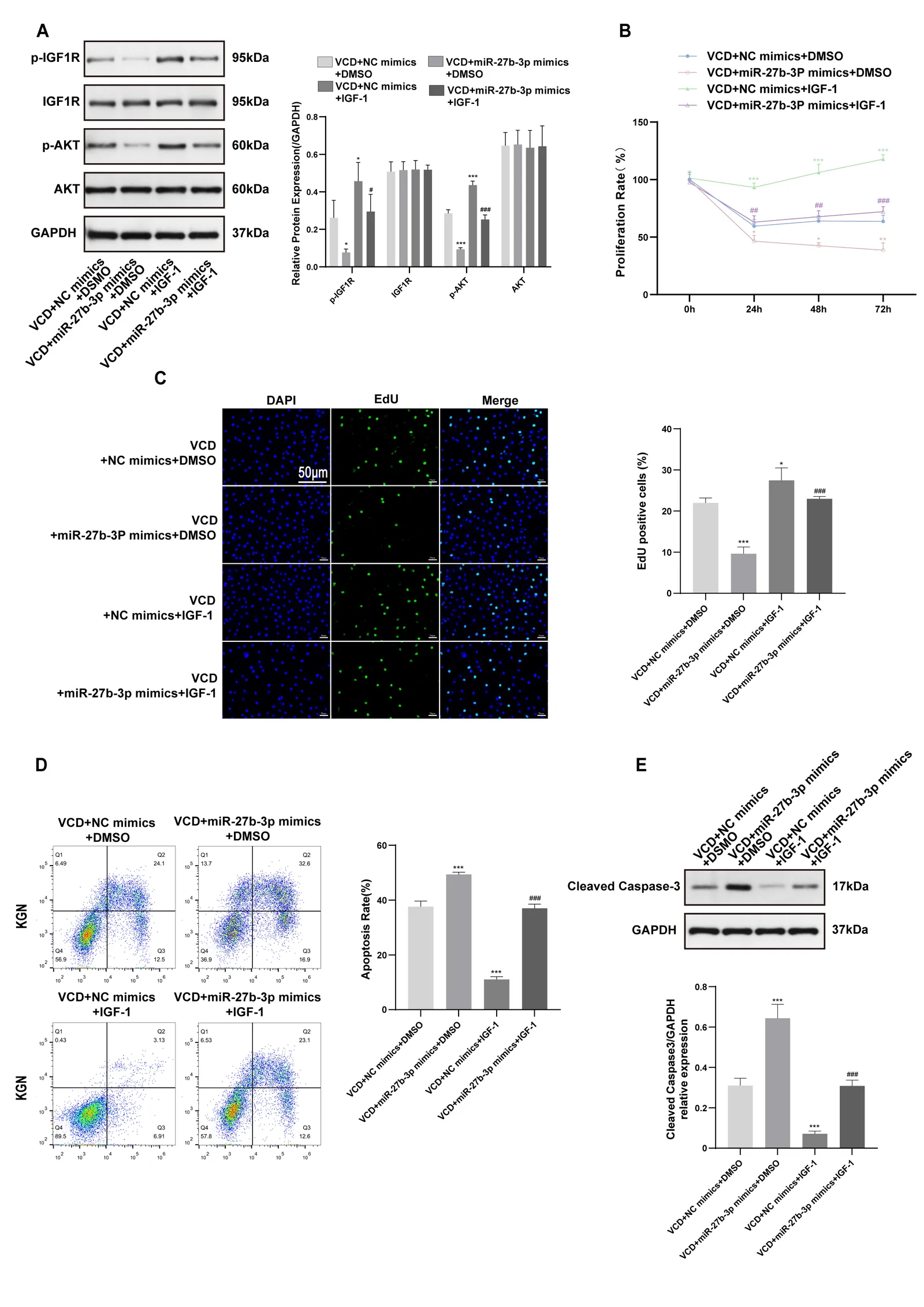
Mechanistic reliance of miR-27b-3p-driven phenotypic shifts on the IGF-1/PI3K/AKT pathway. Following VCD-induced damage and the introduction of miR-27b-3p analogs, KGN cultures were subjected to supplementary interventions utilizing synthetic IGF-1 or a DMSO solvent blank. (**A**) Western blot and quantification of p-IGF1R and p-AKT. (**B**) CCK-8 proliferation assay. (**C**) EdU staining. Scale bar = 50 μm (×200). (**D**) Flow cytometric apoptosis analysis. (**E**) Western blot of cleaved caspase-3. Data represent mean ± SD. * *p* < 0.01, ** *p* < 0.01, *** *p* < 0.001 vs. VCD + NC mimics + DMSO; ^#^ *p* < 0.05, ^##^ *p* < 0.01, ^###^ *p* < 0.001 vs. VCD + miR-27b-3p mimics + DMSO.

To validate whether these phenotypic shifts were mechanistically dependent on the specified pathway, VCD-damaged KGN cells overexpressing miR-27b-3p were supplemented with external IGF-1 (or a DMSO vehicle control). Western blotting confirmed that IGF-1 supplementation specifically restored p-IGF1R and p-AKT levels suppressed by miR-27b-3p (*p* < 0.001; Figure 6A). Functionally, CCK-8 and EdU assays demonstrated that IGF-1 effectively reversed miR-27b-3p–induced proliferation inhibition (*p* < 0.001; Figure 6B,C). Similarly, flow cytometry and Western blotting showed that IGF-1 markedly attenuated the elevated apoptosis and cleaved caspase-3 expression caused by miR-27b-3p (*p* < 0.001; Figure 6D,E). Collectively, these results definitively establish that miR-27b-3p exacerbates VCD-induced granulosa cell injury by depleting local IGF-1 bioavailability and inhibiting PI3K/AKT pathway activation—providing mechanistic validation of the miR-27b-3p → PAPPA → IGF-1 → PI3K/AKT axis in POI pathogenesis.

## 4. Discussion

Characterized essentially by accelerated follicular attrition and aberrant programmed death of granulosa cells (GCs), premature ovarian insufficiency (POI)—previously classified as POF—is a devastating endocrine condition. Clinically, it fundamentally deprives women of childbearing age of their fertility while concurrently triggering a spectrum of prolonged physiological morbidities [14]. In recent years, identifying molecular targets capable of protecting GCs from injury has emerged as a pivotal strategy to halt POI progression or even restore ovarian function [14]. Moreover, modulating the local cellular microenvironment is increasingly recognized as critical for tissue repair and remodeling in female reproductive disorders [21]. 4-Vinylcyclohexene diepoxide (VCD), a well-established environmental ovarian toxicant, selectively depletes primordial and primary follicles by inducing GC apoptosis and autophagy; its efficacy in recapitulating human POI has been validated in rodent models [22]. Building upon this pathophysiological foundation, we successfully established a VCD-induced in vitro injury model using human KGN cells and, for the first time, demonstrated that miR-27b-3p is markedly upregulated under VCD stress. Crucially, we reveal that miR-27b-3p exacerbates GC dysfunction and apoptosis via the PAPPA–IGF-1–PI3K/AKT axis, providing a novel epigenetic mechanism underlying POI pathogenesis.

Recognized as fundamental orchestrators of cellular growth and programmed death, miRNAs exert their regulatory power post-transcriptionally by targeting the 3′UTRs of specific mRNAs, thereby triggering either transcript clearance or translational silencing. This potent regulatory capacity is distinctively illustrated by the ability of miR-182-5p to substantially impede vascular smooth muscle cell proliferation [23]. Accumulating evidence positions miR-27b-3p as a highly active modulator in diverse pathological contexts: it is upregulated in degenerating intervertebral discs, driving cellular senescence [24], and promotes disease progression in prostate cancer through fine-tuned regulation of specific oncogenic targets [25]. Consistent with these observations, our gain- and loss-of-function experiments unequivocally show that miR-27b-3p overexpression severely exacerbates VCD-induced proliferation arrest and apoptosis in KGN cells, whereas its inhibition confers robust cytoprotection. This confirms miR-27b-3p as a critical pro-pathological factor in ovarian granulosa cell aging.

Elucidating the downstream mechanism of miR-27b-3p constitutes the core of this study. Using dual-luciferase reporter assays, we definitively identify PAPPA (pregnancy-associated plasma protein A) as a direct target of miR-27b-3p. PAPPA is indispensable for ovarian physiology, particularly folliculogenesis [26]. As a secreted metalloproteinase, PAPPA specifically cleaves insulin-like growth factor-binding proteins (e.g., IGFBP4), thereby liberating bioactive, free IGF-1 [27]. Extensive studies establish that the IGF/PAPPA regulatory axis is a cornerstone of cell survival within local tissue microenvironments [28]. Clinically, PAPPA levels correlate strongly with local free IGF-1 concentrations—not systemic total IGF-1—highlighting its paracrine/autocrine significance [29]. Collectively, these data explain our key finding: miR-27b-3p, by suppressing PAPPA expression in KGN cells, impedes IGFBP proteolysis, resulting in diminished free IGF-1 availability and consequent collapse of pro-survival signaling.

At the systems level, PAPPA intersects with canonical aging pathways, including Sirtuin-related networks [30], and global Pappa knockout mice exhibit complex, tissue-specific aging phenotypes [31]. Crucially, the biological impact of the IGF-1 network on aging exhibits remarkable spatial plasticity. While organism-wide downregulation of insulin/IGF-1 cascades is widely recognized to delay senescence in laboratory species [32], a contrasting dynamic emerges within hormone-driven microenvironments. Specifically, in targets like ER-positive breast carcinomas [33] and ovarian follicles, heightened PAPPA activity actively safeguards cellular viability and drives localized expansion. Furthermore, inflammatory and oxidative stress potently modulate PAPPA expression in a tissue-specific manner [34]. Thus, in the context of VCD-induced ovarian toxicity, PAPPA downregulation does not confer “anti-aging” benefits; rather, it deprives GCs of essential IGF-1 trophic support, directly driving them toward apoptotic demise.

IGF-1–mediated survival signaling is critically dependent on IGF1R activation and subsequent PI3K/AKT phosphorylation—a canonical anti-apoptotic cascade [15,16]. Prior work in oncology has shown that miRNAs can modulate IGF1R status via upstream targets, thereby dictating PI3K/AKT pathway activity and cell fate [35]. Similarly, in cardiovascular stress models, the PAPPA–IGF axis acts as a potent cardioprotective mechanism [36,37]. In our study, microenvironmental IGF-1 deficiency—triggered by miR-27b-3p–mediated PAPPA suppression—led to blunted IGF1R and AKT phosphorylation in KGN cells. Importantly, both exogenous IGF-1 supplementation and PAPPA overexpression fully abrogated miR-27b-3p–induced apoptosis and proliferation arrest, establishing a complete mechanistic closed-loop validation.

In summary, our findings suggest that aberrant upregulation of miR-27b-3p may contribute to VCD-associated injury in KGN cells, potentially through modulation of the PAPPA/IGF-1/PI3K/AKT signaling pathway. Restoration of PAPPA or IGF-1 partially rescued the cellular phenotypes observed in this in vitro model, supporting a possible role for this axis in the cellular response to VCD exposure. However, these findings should be interpreted cautiously because they were obtained using an immortalized KGN cell line and an in vitro chemical-toxin exposure model. Whether this pathway contributes to POI in vivo, or whether it can be therapeutically targeted, requires further investigation in primary human granulosa cells, animal models, and clinical samples.

An important limitation of this study is that the conclusions were derived from in vitro experiments using VCD-treated KGN cells. Although these experiments provide mechanistic evidence for the role of the miR-27b-3p/PAPPA/IGF-1 axis in granulosa-cell injury, further studies using primary human granulosa cells and a VCD-induced mouse model of POI are required.

## Figures and Tables

**Table 1:** RT-qPCR primer sequences used in this study.

miR-27b-3p	Forward	CTCGCTTCGGCAGCACAT
	Reverse	AACGCTTCACGAATTTGCGT
U6	Forward	TGCACAGTGGCTAAGTTCTGC
	Reverse	CTCAACTGGTGTCGTGGAGTC
PAPPA	Forward	CATCATCCCTGCCTCTACTGG
	Reverse	GTGGGTGTCGCTGTTGAAGTC
GAPDH	Forward	ACCAAGGTGATAGATCTCAGTGAAG
	Reverse	CTGATCTTTGGTATCAAGCAGCT

## Data Availability

The original contributions presented in this study are included in the article. Further inquiries can be directed to the corresponding author.

## References

[1] Webber L., Davies M., Anderson R., Bartlett J., Braat D., Cartwright B., Cifkova R., Keizer-Schrama D.M.S., Hogervorst E., Janse F. (2016). ESHRE Guideline: Management of Women with Premature Ovarian Insufficiency. Hum. Reprod..

[2] Panay N. (2021). Progress in Understanding and Management of Premature Ovarian Insufficiency. Climacteric.

[3] Polakova M. (2023). Specifics of Infertility Treatment in Women with Premature Ovarian Failure. Ceska Gynekol..

[4] Touraine P., Chabbert-Buffet N., Plu-Bureau G., Duranteau L., Sinclair A.H., Tucker E.J. (2024). Premature Ovarian Insufficiency. Nat. Rev. Dis. Primers.

[5] Rudnicka E., Kruszewska J., Klicka K., Kowalczyk J., Grymowicz M., Skorska J., Pieta W., Smolarczyk R. (2018). Premature Ovarian Insufficiency—Aetiopathology, Epidemiology, and Diagnostic Evaluation. Prz. Menopauzalny.

[6] Song W., Qiu Y.T., Chen L.N. (2023). 4-Vinylcyclohexene Diepoxide Induces Apoptosis by Excessive Reactive Oxygen Species and DNA Damage in Human Ovarian Granulosa Cells. Toxicol. In Vitro.

[7] Zhao X., Mao B., Wang J., Wang H., Ma X., Yang K., Yang Y. (2025). The Regulation of ZAR1 on Apoptosis and Mitophagy in Ovarian Granular Cells and Primary Ovarian Insufficiency (POI) Mice. Reprod. Sci..

[8] Zhang J., Xu Y., Liu H., Pan Z. (2019). MicroRNAs in Ovarian Follicular Atresia and Granulosa Cell Apoptosis. Reprod. Biol. Endocrinol..

[9] Chen X., Jiang X., Liu J., Bai X., Wang X. (2020). *miR*-132 Is Upregulated in Polycystic Ovarian Syndrome and Inhibits Granulosa Cells Viability by Targeting Foxa1. Mol. Med. Rep..

[10] Cao Y., Li W., Wu M.Y. (2022). MiR-383-5p Promotes Apoptosis of Ovarian Granulosa Cells by Targeting CIRP through the PI3K/AKT Signaling Pathway. Arch. Gynecol. Obstet..

[11] Ju W., Zhao S., Wu H., Yu Y., Li Y., Liu D., Lian F., Xiang S. (2024). *miR*-6881-3p Contributes to Diminished Ovarian Reserve by Regulating Granulosa Cell Apoptosis by Targeting SMAD4. Reprod. Biol. Endocrinol..

[12] Xu J., Jia Y. (2025). miR-361-5p Regulates SLC25A24 to Maintain Mitochondrial Function and Alleviate Granulosa Cell Dysfunction in Diminished Ovarian Reserve. J. Assist. Reprod. Genet..

[13] Wang M., Sun J., Li X. (2018). Functional Characterization of MicroRNA-27a-3p Expression in Human Polycystic Ovary Syndrome. Endocrinology.

[14] Gu H.C., Wang L.F., Zhang Y.W., Zhuo Y.Q., Zhang Z.H., Wei X.Y., Liu Q.W., Deng K.Y., Xin H.B. (2025). Human Urine Stem Cells Protect against Cyclophosphamide-Induced Premature Ovarian Failure by Inhibiting SLC1A4-Mediated Outflux of Intracellular Serine in Ovarian Granulosa Cells. Cell. Mol. Biol. Lett..

[15] Afradiasbagharani P., Hosseini E., Allahveisi A., Bazrafkan M. (2022). The Insulin-like Growth Factor and Its Players: Their Functions, Significance, and Consequences in All Aspects of Ovarian Physiology. Middle East Fertil. Soc. J..

[16] Botkjaer J.A., Poulsen L.L.C., Noer P.R., Grondahl M.L., Englund A.L.M., Franks S., Hardy K., Oxvig C., Andersen C.Y. (2024). Dynamics of IGF Signaling During the Ovulatory Peak in Women Undergoing Ovarian Stimulation. J. Clin. Endocrinol. Metab..

[17] Conover C.A., Oxvig C. (2023). The Pregnancy-Associated Plasma Protein-A (PAPP-A) Story. Endocr. Rev..

[18] Hourvitz A., Kuwahara A., Hennebold J.D., Tavares A.B., Negishi H., Lee T.H., Erickson G.F., Adashi E.Y. (2002). The Regulated Expression of the Pregnancy-Associated Plasma Protein-A in the Rodent Ovary: A Proposed Role in the Development of Dominant Follicles and of Corpora Lutea. Endocrinology.

[19] Kilic D., Guler O. (2021). Serum Pregnancy-Associated Placental Protein-A (PAPP-A) Levels Are Increased in Polycystic Ovary Syndrome (PCOS) in Women with Oligo-Anovulation. BMC Endocr. Disord..

[20] Mørch N.F., Nøhr B., Kirk M., Rode L., Svendsen P.F. (2026). Early Pregnancy Concentrations of Pregnancy-Associated Plasma Protein-A (PAPP-A) and Insulin-like Growth Factor-1 (IGF-1) Following Frozen Embryo Transfer: Secondary Analyses from a Randomized Controlled Trial. Hum. Reprod..

[21] Cao Y., Wu J., Huang J., Fan X., Zhang Y., Li L., Dai Y. (2025). Human embryonic stem cell-derived immunity-and-matrix-regulatory cells promote endometrial repair and fertility restoration in IUA rats. Stem Cell Res. Ther..

[22] Zhou Q., Jin X., Wang J., Li H., Yang L., Wu W., Chen W. (2023). 4-vinylcyclohexene diepoxide induces premature ovarian insufficiency in rats by triggering the autophagy of granule cells through regulating miR-144. J. Reprod. Immunol..

[23] Jin C., Gao S., Li D., Shi X., Hu Z., Wang C., Xiao J., Sheng Z., Ding Z., Zhang D. (2020). MiR-182-5p Inhibits the Proliferation of Vascular Smooth Muscle Cells Induced by ox-LDL Through Targeting PAPPA. Int. Heart J..

[24] Guo W., Mu K., Geng J.C., Xing H.Y., Dong Y., Liu W.D., Wang S.C., Shi J.X., Xing B.R., Zhao J.Y. (2025). ATF1 and miR-27b-3p drive intervertebral disc degeneration through the PPARG/NF-κB signaling axis. Commun. Biol..

[25] Yang L., Ruan Y., Chen B., Zhu Y., Xu H. (2024). Circ_0001671 regulates prostate cancer progression through miR-27b-3p/BLM axis. Sci. Rep..

[26] Schaab A.M., Stroud K.L., Nguyen D.T., Moses J.C., Hart J., Aplin Z.J., Chosed R.J., Fox C.W., Green L.J., LaVoie H.A. (2025). PAPPA’s role in female reproduction. Reproduction.

[27] Monget P., Oxvig C. (2016). PAPP-A and the IGF system. Ann. Endocrinol..

[28] Hjortebjerg R., Høgdall C., Hansen K.H., Høgdall E., Frystyk J. (2024). The IGF-PAPP-A-Stanniocalcin Axis in Serum and Ascites Associates with Prognosis in Patients with Ovarian Cancer. Int. J. Mol. Sci..

[29] Nimptsch K., Aydin E.E., Chavarria R.F.R., Janke J., Poy M.N., Oxvig C., Steinbrecher A., Pischon T. (2024). Pregnancy associated plasma protein-A2 (PAPP-A2) and stanniocalcin-2 (STC2) but not PAPP-A are associated with circulating total IGF-1 in a human adult population. Sci. Rep..

[30] Bi S., Jiang X., Ji Q., Wang Z., Ren J., Wang S., Yu Y., Wang R., Liu Z., Liu J. (2024). The sirtuin-associated human senescence program converges on the activation of placenta-specific gene PAPPA. Dev. Cell.

[31] Li X., Hager M., McPherson M., Lee M., Hagalwadi R., Skinner M.E., Lombard D., Miller R.A. (2023). Recapitulation of anti-aging phenotypes by global, but not by muscle-specific, deletion of PAPP-A in mice. Geroscience.

[32] Bartke A. (2008). Impact of reduced insulin-like growth factor-1/insulin signaling on aging in mammals: Novel findings. Aging Cell.

[33] Mabruk Z., Bullock E., Xiao X., Guo J., Zhu X., Gómez-Cuadrado L., Oxvig C., Mallon E., Ammar A., Malty A. (2026). High Expression of PAPP-A Predicts Poor Outcomes in Oestrogen Receptor-Positive Breast Cancer Patients. Cancer Med..

[34] Li W., Li H., Zhou L., Wang Z., Hua B. (2019). Pregnancy-Associated Plasma Protein A Induces Inflammatory Cytokine Expression by Activating IGF-I/PI3K/Akt Pathways. Mediat. Inflamm..

[35] Han J. (2016). Regulation of miR-223 on the Erlotinib-Resistant PC9 Cell Lines via IGF-1R/PI3K/Akt Bypass. Ph.D. Thesis.

[36] Steffensen L.B., Conover C.A., Oxvig C. (2019). PAPP-A and the IGF system in atherosclerosis: What’s up, what’s down?. Am. J. Physiol. Heart Circ. Physiol..

[37] Basaran K.E., Korkmaz S., Satır-Basaran G., Salkın H. (2024). Short and long-term blockades of adenosine 2A, 5-HT2A, and 5-HT7 receptors induce apoptosis, reduce proliferation, and show differential effects on miR-27b-3p expression in neuroblastoma cell lines. Neuroscience.

